# BCR::ABL1 e1a2 (p190)-Positive Myeloid Blast Neoplasm: Diagnostic Challenges in Distinguishing De Novo Acute Myeloid Leukemia From Previously Unrecognized Chronic Myeloid Leukemia in the Blast Phase

**DOI:** 10.7759/cureus.113606

**Published:** 2026-07-29

**Authors:** Valeria González Quiroz, Raul Sergio Campillo González, Jorge Alberto Garay Hernandez, Guadalupe de los Angeles Salazar Gonzalez, Cesar Fabian Vallejo Rico

**Affiliations:** 1 Internal Medicine, Mexican Social Security Institute, Durango, MEX; 2 Hematology, Mexican Social Security Institute, Durango, MEX

**Keywords:** acute myeloid leukemia (aml), bcr-abl1, bcr-abl transcript, blast cell, chronic myeloid leukemia, e1a2, philadelphia chromosome, tyrosine kinase receptor inhibitors

## Abstract

Chronic myeloid leukemia (CML) is a myeloproliferative neoplasm driven by the BCR::ABL1 fusion gene (abelson breakpoint clustering region 1), usually resulting from the reciprocal translocation t(9;22)(q34;q11.2), which generates the Philadelphia chromosome. Most patients with CML express the p210 BCR::ABL1 protein, typically generated by the e13a2 or e14a2 transcripts. In contrast, the e1a2 transcript, which encodes the p190 BCR::ABL1 isoform, is uncommon in CML and is more frequently associated with Philadelphia chromosome-positive B-cell acute lymphoblastic leukemia. Patients with p190-positive CML often present with atypical clinical features, late diagnosis, rapid progression, and reduced survival. According to the WHO classification, the disease progresses through two phases: a chronic phase and a blast phase. The blast phase (CML-FB) is the most aggressive stage of CML and is associated with a poor clinical prognosis.

We present the case of a 48-year-old woman with a two-year history of chronic anemia of unknown origin who developed a blast myeloid neoplasm expressing BCR::ABL1. Peripheral blood analysis showed a marked myeloid predominance with granulocytic precursors in multiple stages of maturation and 35% circulating blasts. Bone marrow examination revealed 35% myeloid blasts expressing CD34, CD117, and myeloperoxidase, with no evidence of lymphoid differentiation. The markers TdT, PAX-5, CD10, CD14, CD36, CD41, and CD20 were negative. Conventional cytogenetic analysis revealed a normal female karyotype (46,XX), while fluorescence in situ hybridization detected the BCR::ABL1 rearrangement. Standard quantitative reverse transcription polymerase chain reaction, designed to detect common p210 transcripts, was negative, while a qualitative assay identified the rare e1a2 transcript (p190).

The patient received induction chemotherapy for acute myeloid leukemia (AML) with a 7+3 regimen (continuous infusion of cytarabine for seven days plus an anthracycline for three days) in combination with dasatinib; following consolidation therapy with the high-dose cytarabine (HiDAC) regimen (cytarabine 5 g/m²), minimal residual disease (MRD) assessment yielded a positive result of 0.65%. Consequently, a second cycle of HiDAC was administered with the aim of achieving MRD negativity, and allogeneic hematopoietic stem cell transplantation will be evaluated as a potential consolidation strategy.

This case highlights the diagnostic complexity of BCR::ABL1-positive myeloid blast neoplasms. The presence of the e1a2 transcript alone does not allow differentiation of de novo BCR::ABL1-positive AML from CML in the myeloid blast phase. In this patient, the prolonged clinical history of unexplained anemia, the presence of granulocytic precursors at multiple stages of maturation, and the integrated clinicopathological findings suggested a previously undiagnosed CML presenting in the myeloid blast phase; however, the absence of historical samples and a documented chronic phase prevented definitive reconstruction of the disease course. An accurate diagnosis requires the integration of clinical history, peripheral blood and bone marrow morphology, immunophenotype, cytogenetics, and molecular findings.

## Introduction

Chronic myeloid leukemia (CML) is a myeloproliferative neoplasm driven by the BCR::ABL1 fusion gene (abelson breakpoint clustering region 1), which typically results from the reciprocal translocation t(9;22)(q34;q11.2) and generates the Philadelphia chromosome. In most cases, the breakpoint within the BCR gene occurs in the major breakpoint cluster region (M-bcr), typically involving exons e12-e16, whereas the breakpoint in the ABL1 gene generally occurs at exon a2. This molecular rearrangement most commonly produces the e13a2 (b2a2) and e14a2 (b3a2) fusion transcripts, which encode the 210-kDa p210 BCR::ABL1 oncoprotein. Less frequently, the breakpoint occurs within the minor breakpoint cluster region (m-bcr), generating the e1a2 fusion transcript and the corresponding 190-kDa p190 BCR::ABL1 protein. Other atypical fusion transcripts, including e19a2, e2a2, e1a3, e6a2, e13a3 (b2a3), and e14a3 (b3a3), have also been described, although they occur considerably less often [1,2].

The e19a2 transcript encodes the larger p230 BCR::ABL1 isoform. Although the e1a2 transcript may occasionally be detected together with the more common e13a2 or e14a2 transcripts, CML characterized exclusively by e1a2 expression, referred to herein as p190 BCR::ABL1-positive CML, remains an uncommon molecular subtype. The constitutively active BCR::ABL1 tyrosine kinase promotes leukemic cell proliferation, increases cell survival, and disrupts normal hematopoiesis. The introduction of tyrosine kinase inhibitors (TKIs) has substantially improved the prognosis for patients diagnosed in the chronic phase; however, progression to advanced disease, particularly the blast phase, remains associated with significant morbidity and mortality [1-4].

Most patients with CML express the p210 BCR::ABL1 protein, generated by the e13a2 or e14a2 transcripts. Less common variants of the BCR::ABL1 transcript include e1a2, which encodes the p190 isoform, and e19a2, which encodes the p230 isoform. The e1a2 transcript is most frequently associated with Philadelphia chromosome-positive B-cell acute lymphoblastic leukemia, but it has also been described, albeit rarely, in CML. Patients with p190-positive CML may present with atypical clinicopathological features. Confirmed cases of p190-positive CML are rare and have been reported to present with atypical clinicopathological features, such as less pronounced leukocytosis, increased risk of progression to the blast phase or early progression to it, and suboptimal responses to TKIs [5-8].

The presence of BCR::ABL1 e1a2 in a myeloid blast neoplasm presents a significant diagnostic challenge. While the detection of BCR::ABL1 is characteristic of CML, the presence of this fusion, including the e1a2 transcript, does not, on its own, distinguish blast-phase CML from de novo acute myeloid leukemia (AML) with BCR::ABL1 expression. This distinction requires the integration of clinical history, peripheral blood and bone marrow morphology, immunophenotype, cytogenetic findings, molecular characteristics, and, when available, evidence of a pre-existing chronic-phase clone [1,9,10].

Diagnosis can be particularly challenging when the patient has no documented history of chronic-phase CML, when conventional cytogenetics does not show a morphologically visible Philadelphia chromosome, or when standard quantitative assays are primarily designed to detect common p210 transcripts. In such cases, qualitative molecular assays capable of detecting atypical BCR::ABL1 transcripts may be essential for diagnosis [11,12].

According to the National Comprehensive Cancer Network (NCCN) guidelines, the treatment of blast-phase CML should be individualized based on the blast crisis lineage, the patient's prior exposure to TKIs, the BCR::ABL1 kinase domain mutation status, and eligibility for allogeneic hematopoietic stem cell transplantation (allo-HSCT). For patients with myeloid blast-phase CML who are eligible for intensive therapy, current recommendations support combining a potent TKI with AML-type intensive chemotherapy, followed by consideration of allo-HSCT in responding patients. Induction may be based on conventional anthracycline-cytarabine regimens, such as 7+3, while other intensive cytarabine-containing regimens may be considered according to institutional practice and patient-specific factors [11,12].

We present an unusual case of BCR::ABL1 e1a2 (p190)-positive myeloid blast neoplasia in a patient with a history of anemia of unknown origin, no documented history of chronic-phase CML, a normal conventional karyotype, and a negative standard quantitative assay for the p210 BCR::ABL1 common transcripts. This case illustrates the diagnostic difficulty of distinguishing previously unrecognized CML presenting in the myeloid blast phase from de novo BCR::ABL1-positive AML.

## Case presentation

A 48-year-old woman with a medical history of type 2 diabetes, hypertension, and recurrent chronic cystitis was referred to our hematology department for evaluation and management of a BCR::ABL1-positive myeloid blast neoplasm. She had no personal or family history of hematologic malignancies and no known exposure to ionizing radiation, cytotoxic agents, or other recognized leukemogenic factors.

Approximately two years before referral, persistent normocytic anemia was incidentally identified during follow-up in the urology department. The patient was subsequently evaluated by a hematologist in private practice. According to the available clinical history, an initial bone marrow aspiration resulted in a dry tap, which reportedly raised the possibility of a fibrotic bone marrow process, including primary myelofibrosis. However, the original bone marrow aspirate, biopsy, peripheral blood smears, pathology reports, and any associated cytogenetic or molecular studies were unavailable for review. Consequently, the morphologic and laboratory findings that led to the initial diagnostic consideration of primary myelofibrosis could not be independently verified.

A subsequent bone marrow biopsy, also performed and interpreted in the same private practice setting, was reportedly considered compatible with myelodysplastic syndrome. However, the original pathology report and histologic material were not available for independent review. The specific subtype of myelodysplastic syndrome, the degree and distribution of dysplasia, the bone marrow blast percentage, cytogenetic findings, and any molecular studies performed at that time were unknown. Therefore, the previous diagnosis of myelodysplastic syndrome could not be confirmed according to current diagnostic criteria. The patient was managed conservatively with clinical observation and periodic laboratory monitoring and did not receive disease-specific therapy.

The patient was subsequently referred to our institution because of progressive hematologic abnormalities and clinical deterioration. On evaluation, peripheral blood examination demonstrated a myeloid-predominant proliferation with granulocytic precursors at multiple stages of maturation and 35% circulating blasts. The differential count included 35% blasts, 8% promyelocytes, 12% myelocytes, 12% metamyelocytes, 8% band neutrophils, 15% segmented neutrophils, 3% eosinophils, 2% basophils, 3% lymphocytes, and 2% monocytes. The erythroid series showed anemia, while the platelet count was abnormal (142 X 10^3^/mm^3^) (Table 1).

**Table 1:** Laboratory tests

Parameter	October 2024	June 2025	May 2026
Hemoglobin	6.0 g/dL	6.5 g/dL	6.2 g/dL
Hematocrit	20.0 %	23.0 %	18 %
Mean corpuscular volume (MCV)	99.8 fL	97 fL	98 fL
Mean corpuscular hemoglobin (MCH)	30.9 pg	31 pg	32 pg
Reticulocytes	0.5 %	0.3 %	0.4 %
White blood cells	8.25 ×10³/µL	20.00 ×10³/µL	50.00×10³/µL
Lymphocytes	19 %	5 %	3 %
Absolute lymphocyte count	1.57 ×10³/µL	1.00 ×10³/µL	1.50×10³/µL
Monocytes	9 %	3 %	2%
Absolute monocyte count	0.70×10³/µL	0.60×10³/µL	1.00×10³/µL
Eosinophils	0 %	8%	3%
Absolute eosinophil count	0.00 ×10³/µL	1.60 ×10³/µL	1.50×10³/µL
Basophils	0%	10%	2%
Absolute basophil count	0.00 ×10³/µL	2.00×10³/µL	1.00×10³/µL
Segmented neutrophils	52%	42%	15%
Absolute segmented neutrophil count	4.30 ×10³/µL	8.40 ×10³/µL	7.50×10³/µL
Band neutrophils	7 %	8%	8%
Absolute band neutrophil count	0.60 ×10³/µL	1.60 ×10³/µL	4.00×10³/µL
Promyelocytes	8%	2%	8%
Myelocytes	1 %	12%	12%
Absolute myelocyte count	0.08 ×10³/µL	1.80 ×10³/µL	6.00×10³/µL
Metamyelocytes	11 %	10%	12%
Absolute metamyelocyte count	0.91 ×10³/µL	2.25 ×10³/µL	7.50×10³/µL
Blasts	1 %	0%	35%
Absolute blast count	0.08 ×10³/µL	0.00 ×10³/µL	17.50×10³/µL
Total absolute neutrophil count	6.00 ×10³/µL	7.50×10³/µL	11.5×10³/µL
Platelets	140 ×10³/µL	156×10³/µL	142×10³/µL
Lactate dehydrogenase	200U/l	278U/l	280U/l
HBV surface antigen	-	Non-reactive	Non-reactive
Anti-HCV antibody	-	Non-reactive	Non-reactive
Anti-HIV antibody	-	Non-reactive	Non-reactive
Uric acid	-	5g/mg/dL	6.5g/mg/dl
Quantitative BCR::ABL1 p 210 transcript (e13a2/e14a2)	-	-	0.0%
BCR/ABL t(9;22) translocation	-	-	Detected
Transcript subtype	-	-	e1a2
BCR/ABL1 fusion by RT-PCR	-	-	Detected, e1a2 subtype
Quantitative BCR::ABL1/ABL1 p190 transcript (e1a2)	-	-	38.6% (IS)

The patient had severe anemia and thrombocytopenia without severe bleeding manifestations, and lactate dehydrogenase levels remained within the normal range. Physical examination revealed marked mucocutaneous pallor without lymphadenopathy, hepatomegaly, or splenomegaly. No petechiae, ecchymoses, or active bleeding were observed.

**Figure 1:**
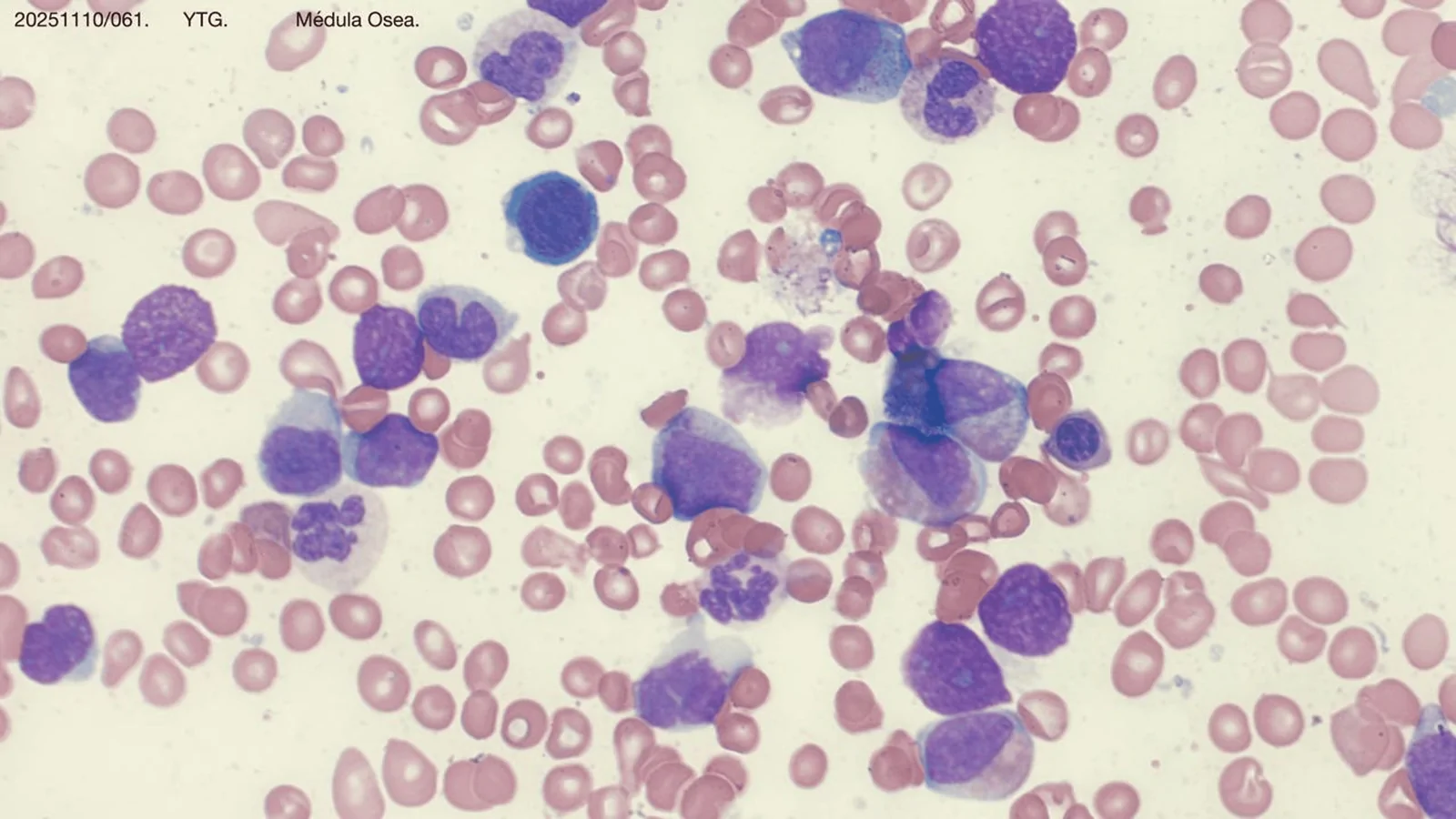
Bone marrow aspirate (Wright-Giemsa stain, 100x magnification) The bone marrow aspirate showed 35% myeloblasts after a differential count of 500 nucleated cells. The blasts are medium to large in size, with a high nucleus-to-cytoplasm ratio, fine and loose chromatin, and one or more visible nucleoli. The cytoplasm is scant or moderately basophilic. Myeloid cells in different lines of differentiation, band neutrophils, metamyelocytes, and myelocytes are also observed.

**Figure 2:**
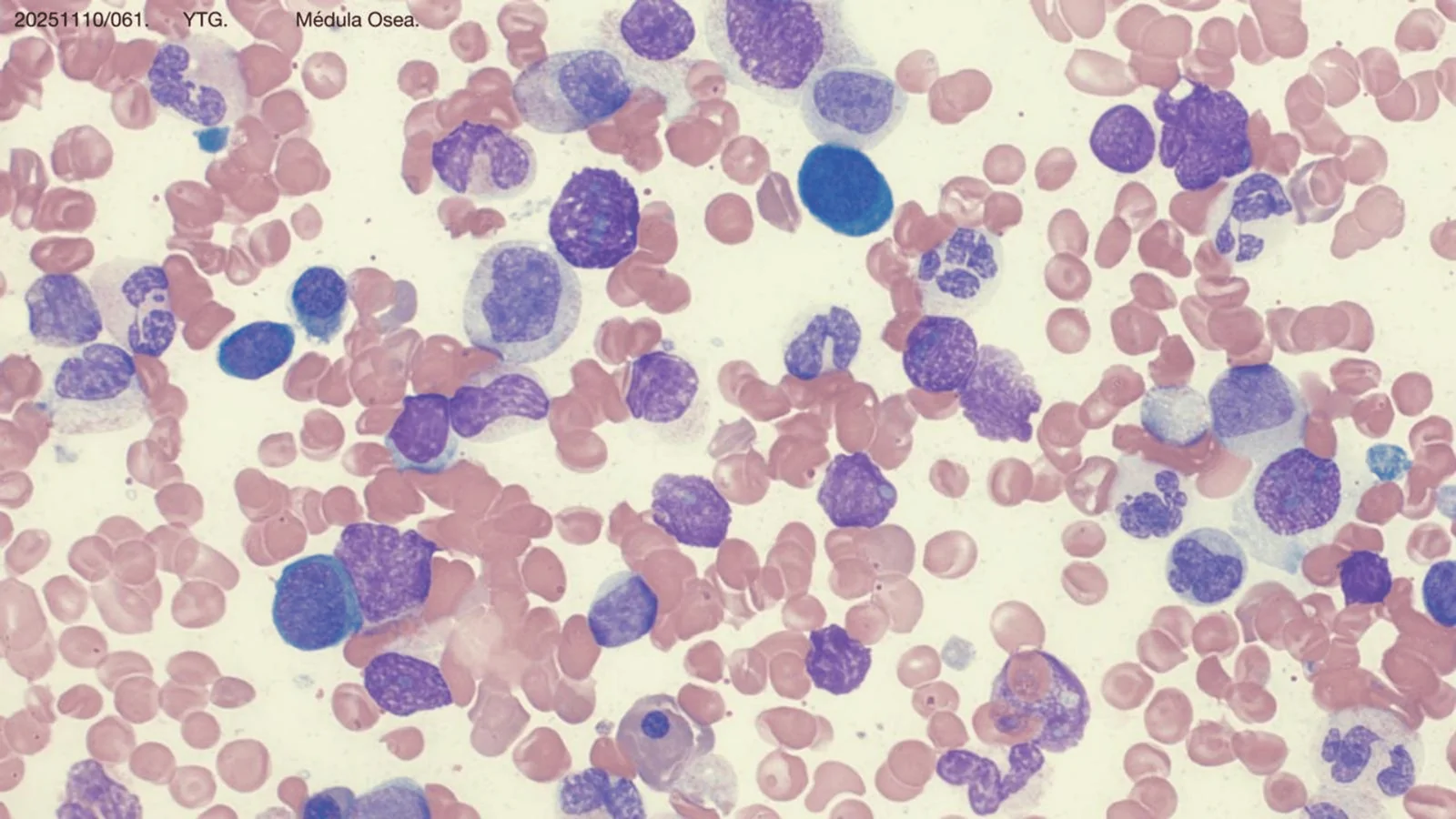
Bone marrow aspirate showing a myeloid blast proliferation with granulocytic maturation at multiple stages. Wright–Giemsa stain, 100× magnification The aspirate is markedly hypercellular and demonstrates a heterogeneous hematopoietic population with a substantial expansion of immature and blast cells. The blasts are medium to large in size, with a high nuclear-to-cytoplasmic ratio, round to oval nuclei, fine to moderately condensed chromatin, and variably prominent nucleoli. The cytoplasm is variably basophilic, with occasional fine azurophilic granulation. Granulocytic precursors at different stages of maturation, including promyelocytes, myelocytes, metamyelocytes, band neutrophils, and segmented neutrophils, are also present. The findings are consistent with a myeloid blast neoplasm with prominent granulocytic maturation.

The Wright-Giemsa-stained bone marrow aspirate showed marked hypercellularity with prominent myeloid proliferation and approximately 35% blasts (Figure 1). The blast population consisted of medium-to-large cells with a high nucleus-to-cytoplasm ratio, fine chromatin, and nucleoli of varying sizes. The cytoplasm was analyzed as moderately basophilic, with occasional fine azurophilic granules (Figure 1). Granulocytic precursors at different stages of maturation were also observed, including promyelocytes, myelocytes, metamyelocytes, band neutrophils, and segmented neutrophils (Figure 2).

**Figure 3:**
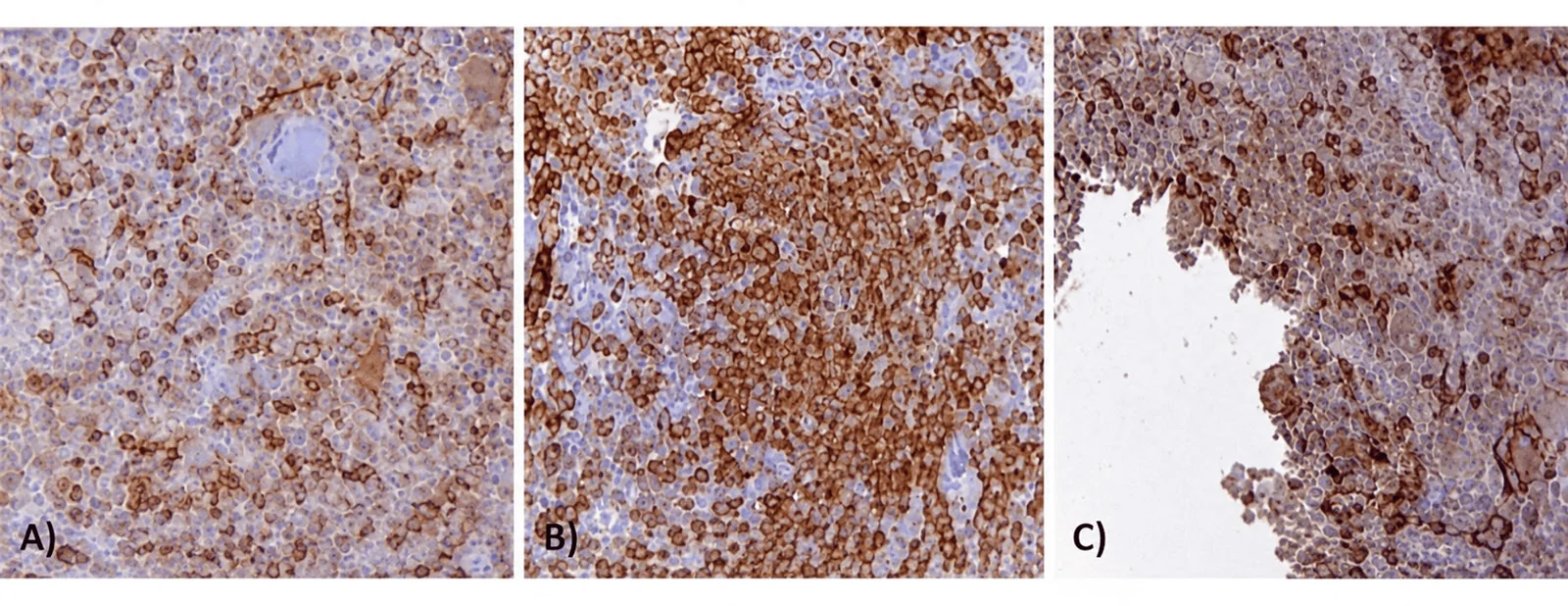
Immunohistochemical study (high-power magnification X100) Histopathological and immunohistochemical study of the bone marrow. A) CD34, B) Myeloperoxidase (MPO), and C) CD117. The blasts showed positivity for MPO, CD34, and CD117. Reticulin staining showed a preserved fibrillar network, without an increase in reticular fibers, corresponding to MF-0 grade bone marrow fibrosis according to the European Consensus Classification System.

Immunohistochemical analysis demonstrated that the blast population expressed CD34, CD117, and myeloperoxidase (MPO). The blasts were negative for terminal deoxynucleotidyl transferase (TdT) and PAX5. Additional evaluated lineage-associated markers, including CD2, CD3, CD5, CD7, CD10, CD14, CD20, CD36, and CD41, were negative. These findings supported myeloid differentiation and did not demonstrate evidence of B-cell or T-cell lymphoblastic differentiation (Figure 3).

Reticulin staining showed a preserved fibrillar network without a significant increase in reticulin fibers, corresponding to MF-0 bone marrow fibrosis. Ki-67 immunostaining showed an estimated labeling index of approximately 15%. This finding was interpreted with caution and was not considered equivalent to the percentage of blasts.

Molecular analysis was performed using a blood smear collected in May 2026; quantitative reverse transcription polymerase chain reaction (RT-qPCR) was conducted to detect the common transcripts p210 BCR::ABL1, e13a2 (b2a2), and e14a2 (b3a2) using a standardized assay with an approximate sensitivity of 0.01% on the International Scale (IS), corresponding to an approximate detection rate of 10⁻⁴. The assay included appropriate internal quality controls for RNA integrity and amplification, as well as positive and negative controls. The p210 BCR::ABL1 assay was negative (0.0%) on the International Scale. 

This result indicated that the assay did not detect the common p210 transcripts but did not exclude the presence of alternative BCR::ABL1 transcripts, including e1a2 (p190), depending on the transcript coverage and validation characteristics of the assay. RT-qPCR was performed on p190, with a result of 38.6%. It had an analytical sensitivity of approximately 10⁻⁴. Internal quality controls included an RNA amplification control, appropriate positive and negative controls, and a reference gene control to confirm sample suitability and the integrity of the extracted RNA.

Due to the negative result for the common p210 transcript and the clinical, morphological, and immunophenotypic findings, further molecular testing was performed using a qualitative RT-PCR assay capable of detecting alternative variants of the BCR::ABL1 transcript. This assay identified the rare e1a2 transcript, corresponding to the p190 isoform of BCR::ABL1. The assay evaluated multiple variants of the BCR::ABL1 transcript, including e1a2 and other rare transcript types.

Fluorescence in situ hybridization (FISH) demonstrated the presence of the BCR::ABL1 rearrangement corresponding to t(9;22)(q34.1;q11.2). In contrast, conventional cytogenetic analysis revealed a normal female karyotype, 46,XX, evaluated in all metaphases, with no morphologically visible Philadelphia chromosome or other chromosomal abnormalities. These findings demonstrated that the absence of a morphologically recognizable Philadelphia chromosome in conventional cytogenetic analysis did not preclude the presence of a BCR::ABL1 rearrangement detected by molecular and FISH-based methods.

The integrated findings confirmed the presence of a BCR::ABL1 e1a2-positive myeloid blast neoplasm. However, because the patient had no prior documented diagnosis of chronic-phase CML, historical samples were unavailable for retrospective molecular testing, and the existence of a pre-existing BCR::ABL1-positive chronic-phase clone could not be demonstrated; therefore, the progression of chronic-phase CML to the blast phase could not be proven with absolute certainty. Consequently, the primary diagnostic considerations were BCR::ABL1-positive AML and previously undiagnosed CML presenting in the myeloid blast phase.

The prolonged history of unexplained anemia, the presence of granulocytic precursors at multiple stages of maturation, the prominent myeloid proliferation, and the overall clinicopathologic context favored previously unrecognized CML presenting in the myeloid blast phase. However, the absence of historical specimens and a documented chronic phase represented an important limitation of the diagnostic assessment.

The patient received induction chemotherapy with a standard 7+3 regimen, consisting of continuous intravenous infusion of cytarabine at 100 mg/m²/day for seven days and doxorubicin at 60 mg/m²/day for three days, combined with dasatinib at a dose of 140 mg orally once daily. Due to the unavailability of daunorubicin, doxorubicin was used as the anthracyclic component.

Following induction therapy, the patient received consolidation with high-dose cytarabine at a dose of 5 g/m². At the time of the last follow-up, two cycles of cytarabine had been administered. Treatment-related toxicities included chemotherapy-induced myelosuppression, which occurred predominantly between days 5 and 10 post-chemotherapy.

Bone marrow evaluation after induction chemotherapy showed persistent disease, with 20% myeloblasts and a detectable minimal residual disease (MRD) of 0.65%. At the time of the last follow-up, the patient was being evaluated for a third cycle of cytarabine, with the aim of achieving MRD negativity and proceeding to allo-HSCT, which was considered the definitive therapeutic strategy after remission.

## Discussion

The present case illustrates the diagnostic complexity of a BCR::ABL1 e1a2-positive myeloid blast neoplasm. The patient had 35% circulating and bone marrow blasts with clear myeloid differentiation, including expression of CD34, CD117, and MPO, without evidence of B-cell or T-cell lymphoblastic differentiation. The detection of the e1a2 transcript was therefore not associated with a lymphoid neoplasm in this patient. Instead, the principal diagnostic challenge was to distinguish between previously unrecognized CML presenting in the myeloid blast phase and de novo BCR::ABL1-positive AML [5,11-14].

The e1a2 transcript is uncommon in CML. In a large series of patients with CML, p190 BCR::ABL1 was identified in a small minority of cases and was associated with atypical clinical characteristics and inferior outcomes compared with the more common p210 BCR::ABL1 transcripts [5]. Subsequent studies have further emphasized that p190 BCR::ABL1-positive CML may represent a biologically distinct subgroup with unusual signaling characteristics and therapeutic challenges [6-8].

The presence of the e1a2 transcript alone, however, does not establish a diagnosis of CML. The same transcript may be detected in other BCR::ABL1-positive hematologic neoplasms, particularly Philadelphia chromosome-positive B-cell acute lymphoblastic leukemia. Therefore, the molecular transcript must be interpreted together with the clinical history, morphology, immunophenotype, and cytogenetic findings [5,11-14].

Features that may support an underlying CML include a documented preceding chronic phase, persistent leukocytosis with left-shifted granulopoiesis, basophilia, thrombocytosis, splenomegaly, and additional clonal abnormalities associated with disease progression [1,9,11,12]. In the present case, the prolonged history of unexplained anemia and the prominent granulocytic proliferation with precursors at multiple stages of maturation favored an underlying chronic myeloid neoplasm. Nevertheless, the absence of historical blood and bone marrow specimens prevented definitive demonstration that the BCR::ABL1-positive clone was already present during the earlier period of anemia.

The distinction between CML presenting in the blast phase and de novo BCR::ABL1-positive AML is clinically and biologically important. Although both entities may present with ≥20% myeloid blasts and BCR::ABL1 positivity, the molecular finding must be interpreted in the context of the disease history and evidence of a pre-existing myeloproliferative neoplasm [11-14]. The ICC and WHO-HAEM5 emphasize the integration of morphologic, clinical, cytogenetic, and genomic information in the classification of myeloid neoplasms and acute leukemias [11,12].

The patient’s peripheral blood showed a prominent granulocytic proliferation with precursors at multiple stages of maturation. This finding was considered more compatible with an underlying chronic myeloid neoplasm progressing to the blast phase than with a purely de novo acute leukemia. However, the absence of a documented chronic phase and the unavailability of historical specimens remain important limitations. Therefore, the most appropriate interpretation is that the integrated findings favored previously unrecognized CML presenting in the myeloid blast phase, while de novo BCR::ABL1-positive AML could not be definitively excluded.

The molecular findings further illustrate the importance of assay design. The initial quantitative RT-qPCR assay was designed to detect common p210 BCR::ABL1 transcripts. The result of 0.0% on the International Scale therefore indicated that e13a2 and e14a2 transcripts were not detected by that assay. It did not necessarily exclude the presence of an alternative BCR::ABL1 transcript. Subsequent qualitative RT-PCR identified the e1a2 transcript and quantitative RT-qPCR detected the BCR::ABL1 p190 (e1a2) at 38.6% on the International Scale [15-17].

Atypical BCR::ABL1 transcripts are clinically relevant because standard molecular monitoring methods may fail to detect them. EUTOS recommendations indicate that patients with atypical transcripts should undergo characterization of the specific fusion transcript and should be monitored using an individualized molecular response strategy rather than automatically applying the International Scale [15]. Accordingly, the 0.0% IS result in this patient should not be interpreted as evidence of complete molecular negativity for all BCR::ABL1 transcripts.

The normal conventional karyotype also deserves consideration. Although the patient had a 46,XX karyotype without a morphologically recognizable Philadelphia chromosome, FISH and molecular testing demonstrated the BCR::ABL1 rearrangement. This finding illustrates that a conventional karyotype without an apparent Philadelphia chromosome does not exclude the presence of a BCR::ABL1 rearrangement. Molecular and FISH-based techniques may detect cryptic or otherwise cytogenetically unapparent rearrangements [11,12,17,18].

The immunophenotype was essential for establishing lineage. The blast population expressed CD34, CD117, and MPO, supporting myeloid differentiation, whereas TdT, PAX5, and B-cell-associated markers were negative. These findings argued against Philadelphia chromosome-positive B-cell acute lymphoblastic leukemia. However, immunophenotype alone cannot distinguish myeloid blast-phase CML from de novo BCR::ABL1-positive AML and therefore must be interpreted together with the clinical history and molecular findings [11-14].

The molecular biology of blast-phase transformation is complex. Progression of CML may be associated with acquisition of additional abnormalities affecting cell-cycle regulation, tumor suppression, epigenetic regulation, hematopoietic differentiation, and intracellular signaling. Similarly, BCR::ABL1-positive AML may harbor cooperating genomic alterations that contribute to its biologic heterogeneity [13,14,18]. A comprehensive myeloid next-generation sequencing panel was not performed in this patient because this technology was unavailable at our institution. Consequently, additional cooperating mutations could not be assessed.

The absence of historical specimens represents the principal limitation of this case. Retrospective analysis of previous peripheral blood or bone marrow samples for BCR::ABL1 would have provided the strongest evidence for or against a pre-existing chronic phase. In the absence of such material, the disease trajectory cannot be reconstructed with absolute certainty.

The patient’s clinical presentation was also atypical for classical chronic-phase CML. Instead of marked leukocytosis, prominent basophilia, thrombocytosis, or splenomegaly, the initial clinical course was dominated by unexplained chronic anemia. This atypical presentation may have contributed to the absence of an earlier diagnosis. However, the absence of typical clinical features does not exclude an underlying CML clone, particularly in patients with rare BCR::ABL1 transcript variants [5-8].

The treatment strategy was based on the provisional diagnosis of a BCR::ABL1-positive blast-phase myeloid neoplasm. The patient received intensive induction chemotherapy in combination with dasatinib. The disease has progressed, and MRD negativity has not yet been achieved. The patient continues with chemotherapy cycles, and once MRD negativity is achieved, allo-HSCT will be evaluated.

Current ELN recommendations support the use of intensive combination chemotherapy with a potent TKI, ideally dasatinib or ponatinib, in eligible patients presenting in or progressing to the blast phase. The therapeutic goal is to achieve a second chronic phase or remission and proceed to allo-HSCT whenever feasible [1,9,10,19]. The 2025 ELN recommendations continue to emphasize the importance of integrating TKI therapy with appropriate acute leukemia-directed treatment and considering transplantation in patients who achieve an adequate response [9].

The use of an AML-type 7+3 induction regimen in this patient was therefore consistent with a lineage-directed intensive chemotherapy approach for a myeloid blast-phase presentation, although treatment selection should be individualized according to disease biology, patient fitness, TKI sensitivity, molecular findings, and institutional protocols [9,10,19].

Overall, the case emphasizes that the detection of the BCR::ABL1 e1a2 transcript in a myeloid blast neoplasm should be regarded as a diagnostic clue rather than as a diagnosis in itself. The final classification requires integration of clinical history, peripheral blood and bone marrow morphology, blast lineage, immunophenotype, cytogenetics, molecular transcript characterization, and, when available, retrospective analysis of previous specimens and comprehensive genomic profiling [11-18].

## Conclusions

BCR::ABL1 e1a2 (p190) positive myeloid blast neoplasms are rare and pose significant diagnostic challenges, especially when there is no documented chronic phase to distinguish de novo BCR::ABL1-positive AML from CML in the blast phase. In this case, the prolonged history of unexplained anemia, the characteristic morphology of the peripheral blood and bone marrow, the myeloid immunophenotype, and the detection of the BCR::ABL1 e1a2 transcript supported the diagnosis of previously unrecognized CML presenting in the blast phase, although definitive confirmation of disease progression could not be achieved due to a lack of historical samples. This case underscores the importance of integrating clinical, morphological, immunophenotypic, cytogenetic, and molecular findings when evaluating BCR::ABL1-positive myeloid blast neoplasms. A negative result on the standard quantitative test targeting only the common p210 transcript should not rule out a BCR::ABL1-associated neoplasm when a high clinical suspicion persists; instead, further testing for atypical transcript variants, such as e1a2 (p190), should be performed. Accurate classification is critical, as it directly influences treatment selection, including TKI therapy, intensive chemotherapy, and consideration of allo-HSCT.
